# Genome Sequence of *Arcobacter* sp. Strain LA11, Isolated from the Abalone *Haliotis discus*

**DOI:** 10.1128/genomeA.00032-17

**Published:** 2017-03-16

**Authors:** Yukino Mizutani, Reiji Tanaka

**Affiliations:** Laboratory of Marine Microbiology, Mie University, Tsu, Mie, Japan

## Abstract

*Arcobacter* sp. strain LA11 was isolated from the gut of the abalone *Haliotis discus*. Here, we present the annotation and analysis of the draft genome of this strain, which is involved in nitrogen metabolism.

## GENOME ANNOUNCEMENT

*Arcobacter* sp. strain LA11 was isolated from the gut of the abalone *Haliotis discus* collected from Mie prefecture, Japan. Phylogenetic trees based on 16S rRNA gene sequences placed the isolates in the genus *Arcobacter*, with *A. bivalviorum* as its closest neighbor. The similarity of the 16S rRNA genes between isolate LA11 and *A. bivalviorum* was 95.3%. On the basis of its phylogenetic and genetic distinctiveness, LA11 was considered to represent a novel species of the genus *Arcobacter*.

*Arcobacter* spp. are widely distributed in the natural environment. In particular, some *Arcobacter* spp. isolated from livestock and humans are pathogenic strains, and the genomes of these strains have been reported (1–4). Recently, many *Arcobacter* spp. have been isolated from marine environments such as seawater, sediment, and shellfish. Among them, the genomes of *A. nitrofigilis* and *A. anaerophilus* have been analyzed (5, 6). *A. nitrofigilis* is associated with the roots of mangroves and contributes to nitrogen fixation (7). Here, we report the first genome sequence of *Arcobacter* sp. LA11, which is involved in nitrogen metabolism.

The genome sequence of *Arcobacter* sp. strain LA11 was completed using paired-end sequencing on an Illumina HiSeq 2500 platform with a HiSeq SBS kit version 4-HS. A total of 52,749,226 paired-end reads comprising 5,274,922,600 bp were obtained. Sequences were pooled and *de novo* assembled using Edena version 3 (8) to reveal a total of 3,098,976 bp with an average G+C content of 27.9% and consisting of 53 contigs. Automated annotation was performed using the Rapid Annotations using Subsystems Technology (RAST) annotation server (9), and overview of the annotated genome was completed with the SEED viewer (10). In addition, rRNA coding and tRNA coding were identified by RNAmmer version 1.2 (11) and tRNAscan-SE (12). The draft genome contains 3,012 coding sequences (CDSs), of which 1,339 CDSs (45%) were classified in 353 subsystems, while 1,673 CDSs (55%) were uncategorized. In addition, 45 predicted noncoding RNAs, including two rRNA genes (SSU:1, LSU:1), 42 tRNA genes for 20 amino acids, and one pseudo-tRNA gene were annotated by the RAST annotation server. However, RNAmmer version 1.2 predicted three rRNA genes (SSU:1, LSU:1, 5S:1) and tRNAscan-SE predicted 43 tRNA genes.

The draft genome revealed the presence of the pathway related to nitrogen metabolism. We also found the *nif* gene cluster (*nifENXZHDKT*), which is involved in nitrogen fixation. In addition, genes encoding for denitrification were present, including the periplasmic nitrate reductase complex (*napAGHBFLD*), nitrite reductase (*nirSNJF*), nitric oxide reductase (*norCB*), and nitrous oxide reductase (*nosYZDFL*). Other *Arcobacter* strains, such as *A. nitrofigilis* and *A. anaerophilus*, had an incomplete denitrification pathway. *Arcobacter* sp. LA11 may have excellent potential for denitrification, and we hope that this report promotes research on the relationship between *Arcobacter* strains and their hosts.

### Accession number(s).

This whole-genome shotgun project has been deposited at DDBJ/EMBL/GenBank under the accession number BDIR00000000. The version described in this paper is the first version, BDIR01000000.
